# Not only Van Gogh: a case of BRASH syndrome with concomitant digoxin toxicity

**DOI:** 10.1186/s13256-024-04600-5

**Published:** 2024-06-09

**Authors:** Ilaria Costantini, Giovanni Mantelli, Massimo Carollo, Lorenzo Losso, Elia Morando, Matilde Bacchion, Mariapaola Castri, Lucia Drezza, Giorgio Ricci

**Affiliations:** 1 USD Poison Control Center, Azienda Ospedaliera Universitaria Integrata, Verona, Italy; 2 Clinical Pharmacology Unit, Department of Diagnostics and Public Health, University of Verona, Verona, Italy; 3 Department of Medicine, University of Verona, Verona, Italy

**Keywords:** Digoxin toxicity, BRASH syndrome, Bradycardia, Hyperkalemia, Case report

## Abstract

**Background:**

Bradycardia, renal failure, atrioventricular (AV) node blocking, shock, and hyperkalemia syndrome is a potentially life-threatening clinical condition characterized by bradycardia, renal failure, atrioventricular (AV) node blocking, shock, and hyperkalemia. It constitutes a vicious circle in which the accumulation of pharmacologically active compounds and hyperkalemia lead to hemodynamic instability and heart failure.

**Case presentation:**

A 66-year-old Caucasian female patient was admitted to the emergency department presenting with fatigue and bradycardia. Upon examination, the patient was found to be anuric and hypotensive. Laboratory investigations revealed metabolic acidosis and hyperkalemia. Clinical evaluation suggested signs of digoxin toxicity, with serum digoxin concentrations persistently elevated over several days. Despite the implementation of antikalemic measures, the patient’s condition remained refractory, necessitating renal dialysis and administration of digoxin immune fab.

**Conclusion:**

Bradycardia, renal failure, atrioventricular (AV) node blocking, shock, and hyperkalemia syndrome is a life-threatening condition that requires prompt management. It is important to also consider potential coexisting clinical manifestations indicative of intoxication from other pharmacological agents. Specifically, symptoms associated with the accumulation of drugs eliminated via the kidneys, such as digoxin. These manifestations may warrant targeted therapeutic measures.

## Background

Bradycardia, renal failure, atrioventricular (AV) node blocking, shock, and hyperkalemia (BRASH) syndrome is a potentially life-threatening clinical condition characterized by bradycardia, renal failure, atrioventricular (AV) node blocking, shock, and hyperkalemia [1]. It encompasses cases where the standard treatment and outcomes significantly deviate from what is expected on the basis of the individual characteristics of the syndrome. This deviation suggests a synergistic effect among the key dysfunctions associated with the syndrome [2].

From a pathophysiological standpoint, atrioventricular nodal blocking medication may cause bradycardia and hyperkalemia, sustaining a vicious cycle including renal dysfunction that may be precipitated from factors, such as dehydration, the use of potassium-sparing diuretics, amiodarone, or medication up-titration. BRASH syndrome is situated within a continuum that ranges from isolated hyperkalemia to signs of severe overdoses of atrioventricular nodal blockers. Although its exact prevalence is not well documented, emerging evidence suggests that the syndrome might be more prevalent than previously acknowledged [3]. The average age at presentation was found to be 70–80 years old, and the most frequent comorbidities were hypertension, diabetes mellitus, and chronic kidney disease [2, 4]. A recently published systematic scoping review of BRASH syndrome clinical characteristics documented a high heterogeneity in clinical presentation, drugs involved, treatment, and prognosis; the mortality rate was determined to be ~ 6% [3].

Most documented cases of BRASH syndrome involved patients on beta-blockers or calcium channel blockers (CCBs), with only a few reported cases in patients taking digoxin [1–4]. The aim of this case report is to highlight the importance of recognizing the potential for BRASH syndrome in patients using digoxin, as managing these patients is particularly challenging; in particular, since digoxin undergoes renal elimination, a decline in kidney function leads to further accumulation of the drug. In addition, digoxin might exacerbate hyperkalemia and bradycardia, propagating the vicious cycle of BRASH syndrome.

## Case presentation

We present the case of a 66-year-old Caucasian female patient who consulted her general practitioner (GP) during a heatwave owing to worsening fatigue over the past 3–4 days. During the consultation, she was found to be hypotensive (80/40 mmHg) and bradycardic (28 bpm), prompting immediate referral to the emergency department (ED). The patient had a medical history of Osler Weber Rendu syndrome, characterized by pulmonary arteriovenous malformations, and had undergone a left mastectomy for breast cancer 7 years prior, with subsequent negative follow-ups. Moreover, she had a platelet function deficit, resulting in a slight prolongation of prothrombin time (PT) with normal values of activated partial thromboplastin time (aPTT) and was experiencing frequent epistaxis. Her medical history also included hypertension, atrial fibrillation—treated with percutaneous occlusion of the atrial appendage 2 years earlier—mitral valve prolapse, pulmonary hypertension, iron deficiency anemia, recent eradication of *Helicobacter pylori* infection, hepatic cirrhosis, and ascites secondary to congestive hepatopathy attributed to cor pulmonale.

Pharmacological history included digoxin at 0.25 mg once per day, bisoprolol at 5 mg twice per day, ramipril at 2.5 mg once per day, spironolactone at 25 mg once per day, furosemide at 25 mg once per day, and pravastatin at 40 mg once per day. Upon her initial evaluation in the ED, she confirmed adherence to her medication regimen, noting that her last doses of digoxin, ramipril, and bisoprolol were taken 2 hours before arrival. At the objective examination, she exhibited mottled skin, slight hypotension (90/45 mmHg), bradycardia (28 bpm), respiratory distress (respiratory rate of 40 breaths/minute), and an oxygen saturation of 89% on room air. She appeared confused. Additionally, she disclosed being anuric for the past 3 days and experiencing visual phenomena such as “flashing lights and sparkles around objects” over the previous week.

**Fig. 1:**
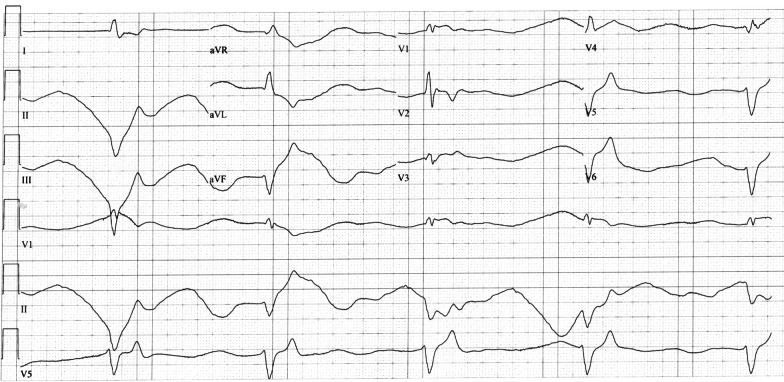
Electrocardiogram on presentation to the emergency department showing an idioventricular rhythm (28 bpm)

The results of the arterial blood gas (ABG) test revealed metabolic acidosis with a pH of 7.10, a pCO_2_ of 32 mmHg, a pO_2_ of 69 mmHg, a bicarbonate level of 10 mmol/L, normal lactate levels, and severe hyperkalemia with a potassium level of 8.9 mmol/L. The initial 12-lead electrocardiogram (ECG) showed an idioventricular rhythm (Fig. 1). She was promptly transferred to the shock room, and treatment was initiated with 10 mL of 10% intravenous calcium chloride, 10 mg of nebulized salbutamol, an infusion of isoproterenol at a rate of 2.5 μg/min, 150 mL of 8.4% intravenous bicarbonate, and 250 mL of 10% dextrose in water (DW10%) with 10 units of insulin.

**Fig. 2:**
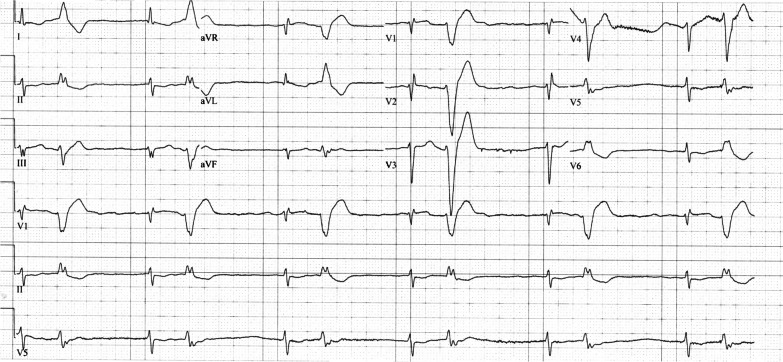
Electrocardiogram on day 1 after normalization of potassium levels. A junctional rhythm with a ventricular bigeminy pattern is seen

**Fig. 3:**
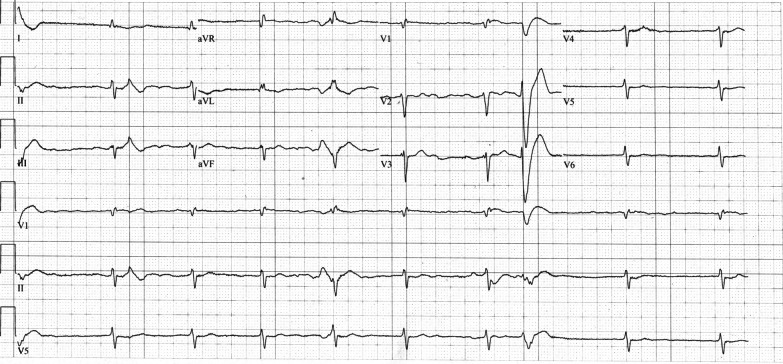
Electrocardiogram on day 3 shows atrial fibrillation with ectopic ventricular beats

**Fig. 4:**
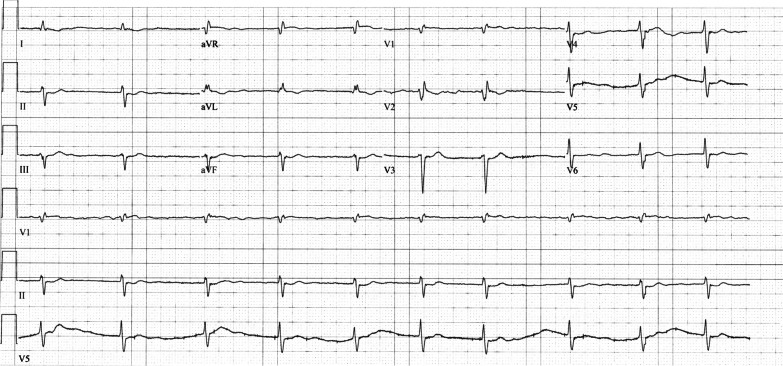
Electrocardiogram on day 14 showing atrial fibrillation returning to baseline rhythm

A subsequent ABG test showed no significant improvement despite potassium-shifting therapy, with potassium remaining high at 9.3 mmol/L. The antikalemic treatment was repeated without success, leading to an urgent consultation with a nephrologist for emergency dialysis. The patient remained bradycardic (30 bpm) and hypotensive (75/50 mmHg) despite isoproterenol infusion. Therefore, the isoproterenol infusion was titrated up to 4 μg/minute aiming to achieve a heart rate of 45–50 bpm. Given the suspicion of concomitant digoxin toxicity, empirical treatment with digoxin immune fab was initiated, administering 40 mg via two vials. Nevertheless, no improvement was observed. Laboratory tests disclosed a serum digoxin level of 13.7 μg/L, a creatinine level of 8.11 mg/dL, and a potassium level of 8.64 mmol/L. After administering an additional five vials of digoxin immune fab, the patient was transferred to the intensive care unit (ICU), where continuous venovenous hemofiltration (CVVH) was promptly initiated, leading to an improvement in serum potassium levels to 4.96 mmol/L the following day. The serum digoxin levels gradually decreased to 1.38 μg/L over 17 days. No further digoxin immune fab administrations were required. The ECGs showed progression, with the normalization of potassium levels and the gradual decrease in serum digoxin. On day 1, the ECG displayed a junctional rhythm with ventricular bigeminy; on day 3, atrial fibrillation with ectopic ventricular beats was observed; and by day 14, the ECG returned to atrial fibrillation (the patient’s baseline; Figs. 2, 3, 4). The corresponding serum digoxin levels are presented in Table 1. After 2 days in the ICU, the patient was transferred to the cardiology ICU, where she continued with CVVH until day 9; the isoproterenol infusion was ceased on day 8. The hospitalization was further complicated by pneumonia and multiple episodes of epistaxis, necessitating blood transfusions.

**Table 1:** Serial blood tests of the patient

	ED arrival	ED arrival (+ 6 h)	Day 1^a^	Day 2	Day 3	Day 5	Day 8	Day 17	Reference level
WBC	10.53	8.19	6.48	5.72	5.29	5.27	5.08	5.84	4.30–10.00 × 10^9^/L
RBC	4.2	2.93	2.76	2.96	3.06	3.06	2.97	3.29	4.00–5.20 × 10^12^/L
HCT	38	25.5	23.9	25.9	0.278	27.5	27.1	29.6	35–47%
Hemoglobin	116	81	7.7	8.5	84	8.5	8.2	9.5	120–150 g/L
MCV	91.9	87.0	86.6	86.9	90.8	89.9	91.6	90.0	80.0–99.0 Fl
MCH	27.6	27.6	27.9	28.5	27.5	27.8	27.7	28.9	26.0–34.0 pg
MCHC	301	318	322	328	302	309	203	321	310–360 g/L
RDW	17.2	17.2	17.6	17.5	17.2	17.0	17.0	18.8	11.5–14.5%
PLT	359	231	201	176	143	100	117	343	150–400 × 10^9^/L
Sodium (Na^+^)	138	139	141	139	139	137	139	143	135–145 mmol/L
Potassium (K^+^)	8.64	4.96	4.33	3.75	3.91	4.09	4.20	3.55	3.40–4.80 mmol/L
Clorum (Cl^−^)		108	106	103					95–107 mmol/L
Calcium (Ca^+^)		2.03		2.36					2.10–2.60 mmol/L
CRP	52		50	83					< 5 mg/L
Creatinine	8.11	5.51	3.81	1.85	1.19	1.26	2.35	5.40	0.49–1.19 mg/dL
eGFR CDK-EPI	4				47	44	20		> 90 mL/min/1.73 m^2^
Urea			16.74	5.35					2.85–7.25 mmol/L
PT (INR)	1.26	1.48	1.56	1.56	1.52				0.80–1.17
APTT ratio	1.14	1.12	1.13	1.25	1.06				0.80–1.20
Digoxin	13.7		10.0	> 10	9.1	5.8	3.8	1.38	0.5–1.0 μg/L
Troponin T	37	29							< 14 ng/L

*APTT* activated partial thromboplastin time, *CRP* C-reactive protein, *ED* emergency department, *eGFR* estimated glomerular filtration rate, *HCT *hematocrit, *INR* international normalized ratio, *MCH* mean corpuscular hemoglobin, *MCHC* mean corpuscular hemoglobin concentration, *MCV* mean corpuscular volume, *PLT* platelets, *PT* prothrombin time, *RBC* red blood cells, *RDW* red blood cells distribution width, *WBC* white blood count

^a^Day 1 corresponds to the day after arrival in the emergency department

The patient was discharged after 35 days in good condition, albeit still requiring intermittent dialysis. High doses of furosemide (250 mg once per day) were also prescribed. Kidney function gradually improved over 3 months, during which the frequency of dialysis was reduced from three times per week to two and then discontinued. At 6 months of follow-up, the patient was diagnosed with stage IIIb chronic kidney disease, had not been hospitalized for renal or cardiological complications, remained in chronic atrial fibrillation (with an average heart rate of 90 bpm), was on therapy with furosemide 250 mg once daily, but did not receive antiarrhythmic or antihypertensive treatment.

## Discussion and conclusion

This particular case involves BRASH syndrome, which led to an accumulation of digoxin. We hypothesize that the syndrome was triggered by initial dehydration, exacerbated by the high temperatures, and worsened by treatment with beta-blockers, digoxin, furosemide, ramipril, and spironolactone. Interestingly, the patient had reported visual disturbances suggestive of digitalis intoxication for over a week. Anecdotally, it is mentioned that Vincent Van Gogh may have produced some of his artworks under the influence of digitalis intoxication [5].

BRASH syndrome is a distinct clinical entity that requires rapid recognition for optimal therapeutic management. Typically, potassium levels in BRASH are not excessively elevated despite often severe bradycardia. In this case, the overlapping digoxin overdose likely exacerbated both hyperkalemia and bradycardia. Furthermore, hyperkalemia likely had a multifactorial etiology, including acute renal failure, dehydration, and medications that the patient was taking as part of their therapy, such as ramipril, spironolactone, and furosemide, in addition to digoxin. In the first few minutes, we administered calcium chloride because of the bradycardia associated with severe hyperkalemia, despite suspicions of possible digoxin toxicity. In our experience, calcium infusion was not associated with arrhythmic complications or diastolic heart failure, contradicting the “stone heart” theory, which lacks robust evidence [6]. Regarding the management of hypotension and bradycardia, a continuous adrenaline infusion could have been beneficial but was not initiated as the patient’s hemodynamics improved with isoproterenol. Isoproterenol was chosen because of its β1 and β2 adrenoreceptor agonist activity as we initially attributed the hypotension to bradycardia and not to a reduction in peripheral vascular resistance. In a subsequent multidisciplinary debriefing, we considered the hyperinsulinemia euglycemia therapy, starting with a 1 UI/kg bolus followed by 0.5–1 UI/kg/hour infusion. This approach could treat bradycardia and hypotension by acting as a positive inotrope and chronotrope while also significantly reducing hyperkalemia. It is particularly effective in cases of bradycardia primarily induced by beta-blockers or CCB accumulation (often suspected by the presence of bradycardia and slight hyperkalemia with no ECG signs of elevated serum potassium levels) [7].

Regarding the concurrent digoxin overdose, mathematical models would have suggested administering 20 vials of digoxin immune fab to antagonize such levels of digoxinemia. However, since it was a chronic intoxication, we aimed to chelate half of the circulating digoxin [8]. It is important to note that serum digoxin levels measured after administration of digoxin immune fab are not reliable indicators of free drug concentration, as the routine immunoassay test does not differentiate between the digoxin bound to antibodies and the unbound fraction. The ECG pattern evolves in synchrony with the serum digoxin concentrations, making ECG a valuable tool for ongoing monitoring [9, 10].

Distinguishing between a case of BRASH syndrome and one of chronic digoxin intoxication can be challenging, as the conditions may potentially overlap. To the best of our knowledge, in the published case report of BRASH syndrome where digoxin was a concomitant drug, the authors found high serum levels of digoxin and classified these instances as chronic intoxication [4, 11–13]. In contrast, BRASH syndrome typically involves beta blockers and calcium channel blockers, for which no routine blood monitoring is currently established [2]. A recently published case report documented, for the first time, the measurement of elevated serum levels of amlodipine in a case of BRASH syndrome [14], lending support to the hypothesis that BRASH syndrome may manifest owing to supratherapeutic drug levels [15, 16]. This observation could explain the persistent bradycardia observed in some patients despite the normalization of potassium levels [17]. In summary, it is possible that BRASH syndrome represents a clinical expression of chronic intoxication attributable to the use of atrioventricular nodal blockers.

In conclusion, early identification of BRASH syndrome is crucial as it is often refractory to standard therapy. The advanced cardiovascular life support (ACLS) bradycardia algorithm may not effectively manage this condition and may lead to mostly unnecessary transvenous pacing. Hyperkalemia often does not respond to potassium shifting, and it then becomes necessary to administer calcium, potassium-wasting diuretics, or contact nephrologists for hemodialysis. Additionally, in many cases, the use of catecholamines is imperative, as fluid resuscitation alone is usually insufficient. Effective treatment of BRASH syndrome involves addressing each contributing factor of the syndrome’s complex cycle. This includes correcting hyperkalemia, ensuring organ perfusion, focusing on hemodynamic stabilization, withdrawing AV nodal blocking agents, and considering dialysis in refractory cases, as any single unaddressed factor could precipitate the vicious cycle. Finally, this syndrome can lead to the accumulation of other drugs subject to renal excretion, such as digoxin, with the ultimate effect of exacerbating the clinical syndrome and other possible toxic effects owing to the overdose itself.

## Data Availability

Data will not be shared because the patient in this case report does not endorse it.

## Abbreviations

ABG: Arterial blood gas
ACLS: Advanced cardiovascular life support
aPTT: Activated partial thromboplastin time
AV: Atrioventricular
BRASH: Bradycardia, renal failure, atrioventricular (AV) node blocking, shock, and hyperkalemia
CCBs: Calcium channel blockers
CVVH: Continuous venovenous hemofiltration
DW10%: Dextrose in water 10%
ECG: Electrocardiogram
ED: Emergency department
GP: General practitioner
H. pylori: Helicobacter pylori
ICU: Intensive care unit
PT: Prothrombin time
